# The Effect of Nordic Walking Training Combined with Vitamin D Supplementation on Postural Control and Muscle Strength in Elderly People—A Randomized Controlled Trial

**DOI:** 10.3390/ijerph15091951

**Published:** 2018-09-07

**Authors:** Jan Mieszkowski, Bartłomiej Niespodziński, Andrzej Kochanowicz, Anna Gmiat, Krzysztof Prusik, Katarzyna Prusik, Jakub Kortas, Ewa Ziemann, Jędrzej Antosiewicz

**Affiliations:** 1 Institute of Physical Education, Department of Anatomy and Biomechanics, Kazimierz Wielki University, 85-091 Bydgoszcz, Poland; mieszkowskijan@gmail.com (J.M.); barnie@ukw.edu.pl (B.N.); 2 Department of Gymnastics and Dance, Gdansk University of Physical Education and Sport, 80-336 Gdansk, Poland; andrzejkochanowicz@o2.pl; 3 Department of Physiology and Pharmacology, Gdansk University of Physical Education and Sport, 80-336 Gdansk, Poland; annagmiat@gmail.com (A.G.); ewann@awf.gda.pl (E.Z.); 4 Department of Recreation and Qualify Tourism, Gdansk University of Physical Education and Sport, 80-336 Gdansk, Poland; krzysztof.prusik@awfis.gda.pl (Kr.P.); prusikkatarzyna@gmail.com (Ka.P.); jakub.kortas@awfis.gda.pl (J.K.); 5 Department of Bioenergetics and Physiology of Exercise, Medical University of Gdańsk, 80-336 Gdansk, Poland; 6 Department of Biochemistry, Gdansk University of Physical Education and Sport, 80-336 Gdansk, Poland

**Keywords:** Vitamin D concentration, high-intensity interval training, Nordic Walking, aging

## Abstract

Nordic Walking (NW) and Vitamin D concentration (Vit D) alone have been shown to contribute to the health and performance of elderly people. However, the interaction between these two factors has yet to be explored. In this study 42 women over 60 years of age (69.02 ± 5.56 years) were recruited and divided in two NW groups: a high-intensity interval training group (HI-NW) and a moderate-intensity continuous training group (MI-NW). Individuals from each group completed a 12-week NW training program (3 times a week/2 hours) combined with randomized Vitamin D supplementation (HD = high dose: 4000 IU/day or LD = low dose: 800 IU/day). Body composition, postural control, muscle strength and Vitamin D serum concentration were measured twice; before and after the intervention. To investigate the interaction between supplementation and training a mixed-design analysis of variance (ANOVA) was performed. The HI-NW group, regardless of supplementation dose, increased their Vit D and elbow torque performance. On the other hand, in the MI-NW group the same Vit D outcome was seen only with HD supplementation and was also associated with increased leg muscle mass. In conclusion, beneficial effects of both HI-NW and MI-NW training regimes were seen. The impact of the dose supplementation on Vit D and body composition was related to the type of NW training.

## 1. Introduction

The development of involution in structural and functional changes in the human body is a natural and inevitable part of the normal aging process. The consequence of these evolving changes is destabilization and dysregulation of the body’s functions ultimately leading to death, the natural and final stage of the aging process. Among numerous changes related to the aging process, a decline in the efficiency of the circulatory, respiratory and nervous systems as well as a slow and continuous loss of muscle mass and strength are the main physiological problems among the elderly [1,2,3]. Furthermore, a progressive increase in body fat and a corresponding decline in muscle mass takes place [4].

Involution processes affecting the nervous system lead to a decrease in the speed of conduction of nerve impulses. In addition, selective denervation of muscle fibers and the intensification of apoptosis of the spinal cord motoneurons occur. This may be one of the reasons for anincreaseing speed of muscle loss, especially in groups of elderly persons leading, a predominantlysedentary lifestyle [5,6,7]. Along with adecrease of body strength, body balance deteriorates. This results directly from involutional changes in the muscle tissue, but also from the impairment of proprioception, neuromuscular coordination and disturbances of other components associated with postural control [8,9]. Combined, a decrease in muscle mass, strength and body balance are associated with an increasing risk of falls and occurrence of injuries, which can affect normal functioning, lead to disability and to a deterioration of the quality of life at anold age [10]. 

Regular physical activity with a certain intensity exerts a positive influence on the functioning of most of the human body systems [11,12]. Among many different forms of physical training, Nordic Walking (NW), due to the use of accessories (poles) which stabilize the step during movement, is an acceptable and often preferred form of physical activity among the elderly. Nordic Walking is classified as full-body activity that combines walking and cross country skiing. If practiced regularly, implementation of a training regime based on the Nordic Walking model, due to the aerobic nature of this kind of effort, is a well-tolerated form of activity that promotes improvement of muscle strength and fitness [13]. A short-term Nordic Walking training can induce positive changes in the knee muscle strength and functional performance in women with a low bone mass; it may also change the concentration of pro-inflammatory markers [14]. Furthermore, movement characteristics in Nordic Walking, specifically pushing against the ground with dedicated poles with each stride, upper and lower body parts, thus helping to improve the functioning of the whole body [15].

In addition, the favourable effect of physical activity on the muscle tissue and body function may be supported by some dietary substances, such as Vitamin D_3_. 25-HydroxyVitamin D (25-OH-D) has a protective effect against muscle degradation, even that induced by aging, and its concentration can be associated with muscle mass and strength [16,17,18]. Most of Vitamin D effects on the muscle mass and its general functions result from the presence of Vitamin D receptors in the muscle tissue [18,19]. At the same time, aging affects the formation of 1,25-dihydroxyVitamin D (1,25(OH)_2_D; calcitriol), thus leading to an age-related decline in renal function [20]. Moreover, 1,25(OH)_2_D serum concentration is dependent on an adequate supply of the substrate for Vitamin D, thus, a developing Vitamin D deficiency leads to a further reduction in 1,25(OH)_2_D concentration. 

A meta-analysis conducted by Muir and Montero-Odasso [21] looking into the effect of Vitamin D supplementation on the muscle strength and the level of body balance in elderly population has revealed that a regulated status of this compound can improve the level of muscle strength and body balance. Such an effect can additionally lower the risk of falls and injuries, especially in the aging population group. In their meta-analysis, Bischoff-Ferrari et al. [22] have demonstrated that the supplementation of Vitamin D reduces the risk of falling in the elderly populations by about 20%. This may contribute to an improved health level in elderly, where falls, fractures and their consequences often contribute to a decline of life quality and contribute various systemic disorders. Compelling data support the efficacy of exercise and Vitamin D status normalization in improving the muscle mass and function in aging populations. Nordic Walking combined with Vitamin D supplementation can, thus, be considered beneficial to elderly’s health, yet the knowledge of training specificity and supplementation doses in this context is still limited [15].

In light of these developments, the aim of this study was to evaluate the impact of the dose of Vitamin D_3_ supplementation and a 12-week programme of Nordic Walking training on the muscle strength and postural control in elderly people, considering also the relation between the impact of the Vitamin D_3_ dose and the type of training applied.

## 2. Materials and Methods

### 2.1. Study Design and Subjects

The study was set as a randomised controlled trial with parallel groups. The study based on a purposeful selection of participants. Participants were screened for inclusion/exclusion criteria by laboratory assistants. They were excluded if they were involved in any structured endurance exercise and/or had participated in resistance exercise during the six months before the study, had a history of cardiac arrhythmia, were suffering from significant hip or knee problems, or showed unstable cardiovascular disease, neuromuscular disease, neurological disease, autoimmune disease, or any neoplasms, peptic ulcers, anaemia or acute hernia, diastolic blood pressure over 100 mmHg. Exclusion criteria also included taking ergogenic levels of nutritional supplements that may have affected the muscle mass or the aerobic capacity (e.g., creatine, β-hydroxy-β-methylbutyrate) or anabolic/catabolic hormone levels (e.g., androstenedione, dehydroepiandrosterone, etc.), smoking and often alcohol consumption (more than once per week) within six months prior to the study. In addition, the Mini-Mental State Examination (MMSE) [23], was performed to evaluate the presence of cognitive disorders in the experimental group (participating in NW training) which could affect the possibility to finish the experimental training and supplementation period. Only the participants, who obtained 27–30 points (lack of cognitive disorders) became involved in the further stages of the study. 

At the beginning, a group of 86 elderly women volunteered to participate in study. They had already taken part in health campaign organised at the Gdansk University of Physical Education and Sport. The group was assessed for eligibility; in effect 44 women were excluded due to either inclusion criteria (*n* = 18), decline to participate (*n* = 21) or other reasons (*n* = 5). Eventually, 42 elderly female volunteers age 69.02 ± 5.57 years were recruited and divided in two Nordic Walking groups: a high-intensity interval training group (HI-NW, *n* = 16) and a moderate-intensity continuous training group (MI-NW, *n* = 26). The allocation was based on results of the Senior Fitness Test [24]. Women obtaining results above the average for the population were included in the HI-NW group with the remaining participants assigned to the MI-NW group. Both groups completed a 12-week Nordic Walking training programme (two hours, three times a week) combined with randomized Vitamin D supplementation (for specific training and supplementation subgroups: 4000 IU and 800 IU Vitamin D_3_). The randomisation for Vitamin D supplementation was performed using a randomization online software (http://www.graphpad.com/quickcalcs/index.cfm) allowing to achieve two equal numbers of participants in the supplementation subgroups [25]. Participants were instructed not to change their life style or diet throughout the experiment. The analysis and training program were completed at the Gdansk University of Physical Education and Sport. 

### 2.2. Ethics Statement

This study was officially approved by the Bioethics Committee for Clinical Research at the Regional Medical Chamber in Gdańsk (KB 26/14). All researchers were obliged to respect the principles of the Helsinki Declaration. Before the study, subjects received a verbal description of the experiment and gave their written, informed consent to participate in the experiment with an option to withdraw at any time for any reason.

### 2.3. Experimental Design

After a standard medical examination and anthropometric measurements with a body composition assessment (bioelectrical impedance analysis or BIA), postural control as well as lower and upper body strength evaluation were performed one week prior to the start of the experiment. The same procedure was performed again after 12 weeks of training. Samples of whole blood for serum isolation and Vitamin D concentration analysis were collected twice: before the beginning of NW training and one day after the last training session. During the 12 weeks of NW training, participants took one of two randomly assigned doses of Vitamin D: (1) a low dose of 800 IU/day (LD) or (2) a high dose of 4000 IU/day (HD). The study follow the single-blinded, whereby neither the participant nor the person administering the supplementation were aware of the dose received. On most of the stages of the experiment the study follow the double-blinded approach, however, authors decided to enrich the manuscript by elbow outcome. Adding elbow outcome occurred after decoding the primary results, therefore the study was indicated as single blinded.

The low dose of Vitamin D was set at 800 IU according to the recommendations of the British Nutrition Foundation [26] and the American Geriatrics Society [27] for people aged 65 years or above. It is recommended that Vitamin D daily supplement is set at the level of 10 micrograms per day (800 IU/day). Due to ethical considerations we did not include a control group in the study design. Instead, we chose to conduct Vitamin D supplementation across the whole group of participants in order to avoid contributing to health deterioration and further Vitamin D deficiencies so common in a moderate climate, especially in the autumn and winter months, when exposure to sunlight for people over 65 years old is considered particularly limited. Low exposure to sunlight contributes to a decrease in the synthesis of Vitamin D active metabolites and results in a decrease of serum 25-hydroxyVitamin D (25(OH)D). The objective of Vitamin D supplementation should be to compensate for insufficient ultraviolet light exposure. As the experiment took place in the period October–January, we have considered our reasoning with respect to Vitamin D supplementation particularly justified. In population groups aged 65 years and older, a long lasting Vitamin D deficiency would be ethically inappropriate and could contribute to deterioration of health, intensification of osteoporosis and various pathological changes. 

A recent review concluded that the safe upper limit for Vitamin D consumption is 10,000 IU per day taken not for longer than 12 weeks [28,29]. Doses above this increase risk of renal calculi formation, especially in patients with absorptive hypercalciuria and end-stage renal disease patients on dialysis [30]. Therefore, in current experimental procedure high doses of Vitamin D was set to 4000 IU/day (HD). 

### 2.4. Exercise Protocol

Both groups completed 12 weeks of NW training, which included 35 training units. During the whole experiment, participants met three times per week (Monday, Wednesday and Friday) in the morning hours. Every training session lasted for 1h and consisted of a 10-min warm-up, 40 min of NW, and a 10-min cool-down. The main NW training session, performed at a continuous moderate intensity (MI-NW group), was conducted at 60–70% of the maximum heart rate—HR (based on the march test results at a distance of 2000 m, monitored using a sport-tester device to ensure a complex cardiovascular control).

The HI-NW group completed the high-intensity interval training session according to the specified protocol after a proper warm-up. A cycle with the applied acceleration: release ratio of 1:2 (a 30-s acceleration going uphill: a 60-s release going downhill) was repeated eight times for a total of about 12 min. It was followed by 28 min of training performed at 70% HR. The whole session ended with a 10-min warm-down to calm the body after the exercise. 

### 2.5. Blood Collection and Analysis

Blood samples were taken in the morning hours (7:00–8:00 a.m.) by a qualified nurse from the antecubital vein using vacutainer tubes. Samples were collected before and three days directly after the 12-week training program. Samples were centrifuged at 1000× G for 15 min and stored at −80 °C pending analysis. Vitamin D metabolite 25-hydroxy D_3_ (25-OH-D_3_) was measured using mass spectrometry combined with high performance liquid chromatography. The high-performance liquid chromatography system was a Transcend TLX turbo flow 2 system attached to a TSQ Quantum Ultra triple quadrupole mass spectrometer (Thermo Fisher Scientific, Waltham, MA, USA).

### 2.6. Body Composition Assessment

In addition to measuring subjects’ height, weight and Body Mass Index (BMI), their body composition was analysed using bioelectrical impedance method of a multi-frequency impedance body composition analyser (In Body 720, Biospace, Seoul, Korea). The following outcomes were analysed: the lean mass of the dominant arm and the leg, the percentage of fat mass, the skeletal muscle mass and the mineral mass. The dominant upper limb was specified as the one used for writing, and the dominant lower limb as the one preferred for kicking a ball. During measurements, participants wore only undergarments and remained barefoot.

### 2.7. Muscle Strength Assessment 

Isometric knee and elbow muscle functions were measured using a Biodex System 4 dynamometer (Biodex Medical Systems, Inc., Shirley, NY, USA). Data collection was performed using a Compaq Desk Pro personal computer and a Biodex software following the standard Biodex protocol. The reliability of analyses performed using a Biodex System 4 dynamometer was on the level of repeatability between good and excellent, depending on the joint evaluated [31,32]. The examined person was familiarised with the specificity of the isometric tension as well as the entire test protocol before recording the torque of the joint. After a standardised warm-up, the subject was positioned according to the manufacturer’s manual. Measurements of the peak torque were taken for flexion (KF) and extension (KE) at the knee joint and for flexion (EF) and extension (EE) at the elbow joint. Measurements of the knee torque were performed with an angular position of 90° in the knee and hip joints, whereas the elbow torque was measured in an angular position of 90° in the elbow and glenohumeral joints. For each joint and activity, the participant performed three trials of a 4-s isometric contraction. Between each trial, a one-minute break was used to properly recover muscle energy. The highest peak torques were taken into analysis. During all measurements, the subject was given verbal encouragement to achieve her maximum potential.

### 2.8. Postural Control Assessment

Postural control was measured in the morning in a quiet indoor laboratory on an AccuGait force platform (Advanced Mechanical Technology Inc., Watertown, MA, USA), recording the displacement of the centre of pressure (COP) using an AMTI software. The measurement of static postural control in the upright position was based on trials on both legs with eyes open (EO) and closed (EC), on a single leg (SL) with eyes open, and in a tandem stance (TS). The SL trial was performed with the dominant leg, which was situated right behind the non-dominant foot during the TS. All subjects were monitored by an observer for safety. During the both legs measurement, the participant’s feet were placed parallel at hip width. For all test trails, measurements were repeated three times. Each trial lasted 30 s, with a frequency sampling of 100 Hz which was low pass filtered at 5 Hz, using a rectangular filter in the frequency domain. During each trial, the subject was asked to stand as still as possible, with the arms at their sides, looking straight forward. The level of body balance was determined by the average velocity (cm·s^−1^) measured as the COP path length divided by the trial time, and the area of the 95th percentile ellipse (95 cm² area) [33,34].

### 2.9. Statistical Analysis

Data were analysed using a two-factor mixed-design analysis of variance (ANOVA). The within-subject factor (repeated measures) represented training effects (pre- and post-measurements), while the between-subject factor showed the effect of the dose of Vitamin D supplementation (HD and LD). Significant interaction between factors were subsequently analysed using Tukey’s post-hoc tests. Two sets of ANOVA tests were performed, one for each type of training (HI-NW, MI-NW). The reliability of assumptions of this statistical test was checked using the Shapiro and Wilk’s test for normality and the Levene’s test for homogeneity of the variance. If the participant’s result exceeded the value of three standard deviations in a given group, it was removed from the given analysis. Due to two separate sets of ANOVA tests, the Bonferroni correction was applied and the level of significance for all analyses was set at *p* < 0.025. To determine the pre/post training effect size, the Cohen’s d statistical calculation was used. The effect size magnitude was categorized as follows: small (d = 0.2–0.59), moderate (d = 0.6–1.19), large (d = 1.2–1.99), very large (d = 2.0–3.99) [35]. Statistical analysis was performed using the Statistica 10 software (Statsoft Inc., Tulsa, OK, USA).

## 3. Results

All 42 participants recruited for the study completed it with no adverse events reported. Characteristics of each subgroup are shown in Table 1. There were no significant differences among each group. Table 2 shows Vitamin D concentration before and after NW training in each subgroup.

In the HI-NW group, both supplementation doses were associated with a significant (pre/post training, *p* = 0.0001) increase of Vitamin D concentration. In the MI-NW group, only the HD supplementation resulted in a significant increase of Vitamin D (*p* = 0.0156). The results of body composition analysis are presented in Table 3. There were three outliers: one in the MI-NW and LD subgroup and two in the HI-NW and HD subgroup.

There were no significant differences between participants’ body weight, body composition and BMI across experimental groups at the beginning of the study. There was no significant main effect or interaction in the HI-NW group. On the other hand, the analysis of the estimated body composition in the MI-NW group showed a tendency in interaction between the supplementation factor and pre/post training measurements in the skeletal muscle mass (*p* = 0.0283) and leg lean mass (*p* = 0.0119). The HD subgroup exhibited a significantly increased SMM in comparison to the LD subgroup (4% vs. −2%, respectively). Similarly, the HD subgroup showed an increased leg lean mass by 4% while the LD subgroup recorded no change. While there was no significant interaction in the arm lean mass (*p* = 0.0808) or the mineral mass (*p* = 0.0534), there was a tendency toward higher values in the MI-NW group receiving HD supplementation.

The analysis of the peak knee torque results showed a significant effect (*p* = 0.0102) of pre/post measurements in KF, where after training, MI-NW participants exhibited increased values for the parameter. What is more, the interaction between the two analysed factors showed that higher peak torque values were seen only in the HD subgroup (Table 4). This result, however, showed only a statistical tendency (*p* = 0.0327). Conversely, for the elbow joint, a significant pre/post effect was seen in the HI-NW group both for EF (*p* = 0.0001) and EE (*p* = 0.0231). Regardless of the supplementation dose, the peak torque of both EE and EF increased by 11%. In the knee torque analysis two outliers in the MI-NW and LD subgroup and one in each of the HI-NW subgroups (LD and HD) were identified. The same outliers in the HI-NW were recognised for the elbow torque, with the additional two in the MI-NW and LD subgroup.

The postural control results showed a significant effect (*p* = 0.0225) of the supplementation on the average velocity of CoP during the EO trial in the HI-NW group. Post-hoc tests of the interaction (*p* = 0.0372) between the supplementation and pre/post measurements showed that after training, the HD subgroup had an 18% lower average velocity of CoP in comparison to the LD subgroup, reflecting, however, only a statistical tendency. In the TL trial, a significant pre/post effect (*p* = 0.0089) was seen, whereas after the MI-NW training, participants had an 11% lower average velocity of CoP. The only effect noticed in Area 95 was that of supplementation (*p* = 0.0229) in the SL trial. This was due to the 64% higher results in the HD group regardless of pre/post measurements. No major effects or interaction were seen in the EC trial of both the HI-NW and MI-NW groups. Detailed results of the postural control test are shown in Table 5.

## 4. Discussion

The study investigated the impact of the 12-week NW training programme with additional supplementation of Vitamin D in elderly women. Previous studies have shown that this kind of training combined with Vitamin D supplementation can provide beneficial effects in terms of total cholesterol, triglycerides, low-density lipoprotein cholesterol [36], pro-inflammatory markers [15] and cognitive functions [37].

The current study focused on the interaction between NW training and Vitamin D supplementation dose in terms of the muscle strength, body composition and postural control. In each of the analysed components, the two different doses of Vitamin D supplementation elicited different outcomes. Participants supplemented with 4000 IU/day of Vitamin D recorded a higher increase in their skeletal muscle mass and leg lean mass than participants supplemented with 800 IU/day of Vitamin D. This change was associated with a higher increase in the KF torque. This outcome is in line with results of Scott et al. [38], who observed a significantly lower body fat and a higher muscle mass at baseline followed by furthermore desirable changes of body composition in elderly adults with high baseline Vitamin D status over the course of a 5-year observation. Still, in this study no changes in the fat mass were observed. Similarly, postural control performance in terms of the average velocity in the eyes open trial showed lower values in the subgroup receiving the higher dose of supplementation. It should be noted that the effect of a prolonged Vitamin D supplementation without training on the knee muscle strength and postural control is still inconclusive [21,39].

While the observed changes were associated with the HD supplementation of Vitamin D, it is probable that NW training played a crucial role in eliciting this overall outcome. It has previously been shown that nine months of 400 IU/day Vitamin D supplementation alone or combined with exercise was insufficient to elicit and modify the beneficial outcome of the exercise itself [40]. This suggests that a low dose of Vitamin D supplementation may not be enough to interact with the exercise effect. Furthermore, a 12-month study investigating the influence of 6500 vs 800 IU/day Vitamin D supplementation on postural control (tandem stance), handgrip and the KE muscle strength has demonstrated that while the higher dose lead to a higher increase of the 25(OH)D concentration, there were no differences in participants’ performance [41]. The authors [41] have explained their outcome by stating that not all participants’ baseline Vitamin D concentration was recognized as insufficient or deficient. However, while participants in our study had documented low Vitamin D levels, the same tests also did not show a significant effect of different doses supplemented. It is possible that effects of a higher supplementation dose are related to specific training loads and testing. These could also be associated with a decreased expression of Vitamin D receptors in elderly people [42]. It has been shown that while a continuous training may not increase Vitamin D concentration, it can elevate the expression of Vitamin D_3_ receptors in the skeletal muscle [43]. Therefore, Vitamin D concentration elicited by supplementation may have had this effect due to an increased amount of muscle receptors. 

In their research, Glade [28] and Vieth [29] have shown that even doses up to 10000 IU taken daily for a long time may not induce the side effects of Vitamin D; hence, this study opted for using high daily doses of Vitamin D supplementation.

The study has also noted that differences caused by supplementation were training-type dependent. While the skeletal muscle mass and leg lean mass increased in the HD supplementation subgroup after the MI-NW training, no differences between supplementation regimes were reported after the HI-NW training. A similar observation has been made in the KF and KE torque changes. It may be explained by a different response of the Vitamin D metabolite 25-hydroxy D_3_ concentration after trainings of different intensities. At the beginning of the study, participants were characterized with 25(OH)D concentration defined as insufficient (21–29 ng/mL) [44]. After the HI-NW training, 25(OH)D concentration increased two-fold with respect to pre-training values regardless of the supplementation dose, while in the MI-NW group, the same increase was observed only with in the HD supplementation subgroup, with the 25(OH)D level in the LD subgroup remaining insufficient. This outcome is in line with a number of studies, which have found a relationship between insufficient 25(OH)D levels and the isometric knee strength [45,46] manifested by a lower muscle strength. Previous studies have also shown that Vitamin D concentrations in elderly people can be modulated by physical activity alone. It has been reported that particularly vigorous activities with high intensity had the strongest positive association with Vitamin D levels, while non-vigorous activities like walking did not [47,48,49]. Moreover, Pilch et al. [50] have demonstrated that six weeks of MI-NW (70% VO_2_ max) was sufficient to decrease the 25(OH)D blood level. It was suggested that an increased metabolic demand of skeletal muscles had a reducing effect on circulating Vitamin D levels. Therefore, it is possible that the contribution of 25(OH)D to muscle metabolism during the MI-NW was higher than in the HI-NW, and thus, only the higher dose of supplementation elicited a notable effect on the lower limb muscle mass and strength.

At the same time, despite the increased Vitamin D concentration in both supplementation subgroups in response to the HI-NW training, no difference in the lower limb performance or muscle mass has been seen. This suggests that a continuous rather than an intermittent type of Nordic Walking training is preferable in inducing the lower limb strength and muscle mass changes in elderly people. Similarly, no relation between the lower limb performance and 25(OH)D concentration has been reported either in the study investigating combined effects of six weeks of HIIT on a cycloergometer and 4000 IU/day Vitamin D supplementation [51]. However, in the aforementioned study [51], the participants’ performance increased regardless of the supplementation or placebo intervention. The lack of effect on the lower limb in the HI-NW group could be related different specificities of the applied training load (Nordic Walking vs. cycling), testing (peak torque vs peak power output and VO_2_ max) as well as differences in participants’ age (19–45 vs. 62–82 years old). Research by Jastrzebska et al. [52] has also shown the same lack of correlation between 5000 IU/day Vitamin D supplementation and an 8-week HIIT training on the lower limb explosive muscle strength in well-trained junior soccer players. 

Nordic Walking training is a physical activity engaging not only lower limbs, but also upper body. Results of the elbow muscle strength similarly have exhibited no relation to the supplementation dose and the HI-NW training as was the case for the knee joint, however, both the peak torque in EF and EE increased in contrast to no change in the peak torque in KF and KE. It is possible that during the HI-NW training, participants engaged more of their upper body to help themselves go uphill in comparison to MI-NW training conditions [53]. Overall, the observed increase in the leg muscle mass, muscle strength and postural control and Vitamin D concentration may contribute to an improved well-being in elderly [54] as well as a reduced risk of falls [55,56] and fractures often resulting from them [22,57].

On the other hand, a recently published review indicated that high dose of supplementation of Vitamin D might increase probability of falls among elderly people [58]. Therefore the review’s author emphasized the need for further investigation into effects of Vitamin D supplementation. In elderly, falls be caused not only by a high level of Vitamin D, but also by low muscle mass and the resulting low level of Vitamin D receptors (VDR). Data on the influence of Vitamin D supplementation on the muscle mass and strength remains ambiguous. We, thus, agree with the need for further research in this area. The study has faced some limitations. Firstly, both NW training modes differed not only in their intensity, but also in the activity of muscle groups engaged; the HI-NW included up- and down-hill walking. Secondly, the allocation to the HI-NW group was based on a better performance in the Senior Fitness Test. Therefore, the lack of difference in the impact of supplementation doses could be partially explained by participant’s slightly better overall condition. However, as baseline 25(OH)D levels in both training groups were similar, this effect is likely to be mainly related to the specificity of training.

## 5. Conclusions

In summary, both the HI-NW and the MI-NW training combined with Vitamin D supplementation were able to elicit beneficial effects in elderly women in terms of an increased muscle mass and strength as well as improved postural control. The observed impact of the supplemented Vitamin D dose on 25(OH)D concentration and body composition was related to the type of NW training. Given our observations, a high dose of Vitamin D supplementation may be necessary to elicit changes in the muscle strength and mass during a moderate-intensity NW training in elderly women with an insufficient 25(OH)D concentration.

## Figures and Tables

**Table 1:** Baseline characteristics of participants with subgroup division.

Variable	High-Intensity Interval Training		Moderate Intensity Continuous Training	
	800 IU/day D_3_ (*n* = 8)	4000 IU/day D_3_ (*n* = 8)	800 IU/day D_3_ (*n* = 13)	4000 IU/day D_3_ (*n* = 13)
**Age (years)**	67.37 ± 6.30	67.63 ± 7.29	69.08 ± 4.87	70.85 ± 4.61
**Body mass (kg)**	67.78 ± 4.52	65.73 ± 4.51	73.47 ± 14.17	70.09 ± 10.16
**Body height (cm)**	163.75 ± 4.56	162.57 ± 5.59	159.58 ± 7.35	163.08 ± 3.79
**BMI (kg·m^-2^)**	25.23 ± 3.47	24.93 ± 2.54	28.77 ± 4.65	26.29 ± 3.21

BMI: Body Mass Index.

**Table 2:** Change of Vitamin D concentration in two different Nordic Walking training programmes (ng/mL).

Dose	High-Intensity Interval Training					Moderate Intensity Continuous Training				
	Before		After		Cohen’s *d*	Before		After		Cohen’s *d*
	Mean ± SD	95% CI	Mean ± SD	95% CI		Mean ± SD	95% CI	Mean ± SD	95% CI	
**LD**	19.06 ± 8.88	11.64–26.49	37.13 ± 12.33 *	26.81–47.43	1.68	21.77 ± 6.63	17.56–25.98	28.20 ± 12.09	20.52–35.88	0.66
**HD**	22.87 ± 6.26	17.08–28.67	48.04 ± 15.08 *	34.09–61.99	2.18	19.70 ± 8.61	14.49–24.90	40.98 ± 12.40 *	33.49–48.48	1.99

LD (low dose): Vitamin D_3_ dose of 800 IU/day, HD (high dose): Vitamin D_3_ dose of 4000 IU/day; CI: confidence intervals, * significant difference between before and after training at *p* < 0.025.

**Table 3:** Body composition results of bioimpedance analysis before and after Nordic Walking training (mean ± SD).

Dose	High-Intensity Interval Training					Moderate-Intensity Continues Training				
	Before		After		Cohen’s *d*	Before		After		Cohen’s *d*
	Mean ± SD	95% CI	Mean ± SD	95% CI		Mean ± SD	95% CI	Mean ± SD	95% CI	
**Body Mass (kg)**										
**LD**	67.78 ± 4.52	58.84–76.71	67.41 ± 11.79	57.56–77.27	0.04	73.47 ± 14.17	64.46–82.47	72.47 ± 14.58	63.21–81.73	0.06
**HD**	65.73 ± 4.51	60.99–70.47	65.63 ± 5.40	59.97–71.30	0.02	70.09 ± 10.16	63.95–76.24	70.60 ± 10.21	64.43–76.77	0.05
**Fat Mass (%)**										
**LD**	32.44 ± 5.42	27.91–36.97	32.23 ± 6.09	27.13–37.32	0.04	39.45 ± 7.36	34.77–44.12	39.79 ± 7.93	34.75–44.83	0.04
**HD**	30.14 ± 6.38	23.44–36.83	30.52 ± 7.19	22.97–38.07	0.06	36.83 ± 5.88	33.28–40.38	35.59 ± 6.22	31.83–39.35	0.20
**Mineral Mass (kg)**										
**LD**	3.21 ± 0.31	2.95–3.47	3.22 ± 0.35	2.92–3.51	0.03	3.05 ± 0.51	2.73–3.38	2.98 ± 0.35	2.76–3.20	0.16
**HD**	3.19 ± 0.29	2.88–3.50	3.20 ± 0.13	2.87–3.54	0.04	3.15 ± 0.28	2.98–3.33	3.24 ± 0.26	3.09–3.41	0.33
**Skeletal Muscle Mass (kg)**										
**LD**	24.67 ± 2.73	22.38–26.95	24.72 ± 3.13	22.11–27.33	0.02	23.82 ± 4.17	21.17–26.47	23.28 ± 3.32	21.17–25.39	0.14
**HD**	24.86 ± 2.47	22.27–27.44	24.89 ± 3.19	21.55–28.24	0.01	23.65 ± 2.16	22.34–24.95	24.69 ± 2.30	23.29–26.08	0.47
**Arm Lean Mass (kg)**										
**LD**	2.33 ± 0.37	2.02–2.64	2.35 ± 0.39	2.03–2.68	0.05	2.35 ± 0.55	1.99–2.69	2.29 ± 0.47	1.99–2.59	0.11
**HD**	2.34 ± 0.25	2.08–2.59	2.34 ± 0.37	1.95–2.73	0.01	2.26 ± 0.27	2.09–2.43	2.37 ± 0.32	2.17–2.56	0.27
**Leg Lean Mass (kg)**										
**LD**	6.94 ± 0.90	6.18–7.69	6.87 ± 0.99	6.04–7.69	0.07	6.65 ± 1.21	5.87–7.41	6.46 ± 0.97	5.84–7.07	0.17
**HD**	7.05 ± 0.88	6.13–7.97	7.06 ± 1.07	5.94–8.19	0.01	6.66 ± 0.81	6.17–7.15	6.95 ± 0.73 *	6.51–7.39	0.38

LD: Vitamin D_3_ dose of 800 IU/day, HD: Vitamin D_3_ dose of 4000 IU/day; * significant difference before and after particular training at *p* < 0.025.

**Table 4:** Results of peak torque before and after two different Nordic Walking trainings.

Variable	High-Intensity Interval Training					Moderate-Intensity Continues Training				
	Before		After		Cohen’s *d*	Before		After		Cohen’s *d*
	Mean ± SD	95% CI	Mean ± SD	95% CI		Mean ± SD	95% CI	Mean ± SD	95% CI	
**KF**										
**LD**	38.01 ± 8.63	30.02–46.00	44.07 ± 14.07	30.43–57.71	0.50	36.21 ± 7.32	31.29–41.13	36.82 ± 8.16	31.33–42.30	0.08
**HD**	36.66 ± 13.89	23.81–49.50	39.21 ± 16.79	23.68–54.75	0.17	36.99 ± 9.58	31.20–42.78	42.82 ± 10.80	36.29–49.34	0.57
**KE**										
**LD**	101.56 ± 21.14	82.01–121.11	111.79 ± 30.67	83.42–140.15	0.39	103.27 ± 23.56	87.44–119.10	98.06 ± 23.46	82.30–113.82	0.22
**HD**	98.81 ± 32	69.15–128.47	94.49–20.69	75.35–113.62	0.16	92.05 ± 23.19	78.04–106.07	94.82–18.02	83.93–105.71	0.13
**EF**										
**LD**	26.56 ± 3.14	23.66–29.46	29.04 ± 3.62 *	25.69–32.39	0.73	24.94 ± 5.46	20.74–29.14	25.33 ± 4.84	21.62–29.05	0.08
**HD**	24.91 ± 3.33	21.83–27.99	27.83 ± 3.95 *	24.17–31.48	0.80	22.97 ± 5.45	19.69–26.27	24.41 ± 2.89	22.66–26.16	0.33
**EE**										
**LD**	22.69 ± 4.24	18.77–26.61	25.19 ± 6.62	19.06–31.31	0.45	18.60 ± 2.93	16.35–20.85	21.60 ± 5.83	17.11–26.08	0.65
**HD**	20.74 ± 3.14	17.84–23.65	23.13 ± 2.99	20.35–25.90	0.78	23.87 ± 10.69	17.41–30.32	21.88 ± 4.23	19.33–24.44	0.24

LD: Vitamin D_3_ dose of 800 IU/day, HD: Vitamin D_3_ dose of 4000 IU/day; CI: confidence intervals, KF: knee flexion; KE: knee extension; EF: elbow flexion; EE: elbow extension, * significant difference between before and after training at *p* < 0.025.

**Table 5:** Results of two different Nordic Walking trainings and supplementations on postural control variables.

CoP	High-Intensive Interval Training								Moderate-Intensity Continuous Training								
	Velocity (cm/s)				Area (cm^2^)				Velocity (cm/s)					Area (cm^2^)			
	Before		After		Before		After			Before		After		Before		After	
Group	Mean ± SD	95% CI	Mean ± SD	95% CI	Mean ± SD	95% CI	Mean ± SD	95% CI	Group	Mean ± SD	95% CI	Mean ± SD	95% CI	Mean ± SD	95% CI	Mean ± SD	95% CI
	Eyes Opened																
LD (*n* = 8)	1.30 ± 0.12	1.20–1.40	1.46 ± 0.19 ^#^	1.30–1.62	0.83 ± 0.22	0.64–1.01	1.28 ± 0.66	0.72–1.83	LD (*n* = 11)	1.27 ± 0.13	1.18–1.36	1.30 ± 0.25	1.14–1.47	1.42 ± 1.24	0.58–2.25	1.41 ± 0.95	0.77–2.05
HD (*n* = 7)	1.29 ± 0.12	1.18–1.40	1.20 ± 0.13	1.09–1.32	1.17 ± 1.02	0.23–2.11	0.79 ± 0.28	0.53–1.05	HD (*n* = 13)	1.41 ± 0.27	1.24–1.57	1.35 ± 0.29	1.18–1.52	1.01 ± 0.72	0.58–1.44	1.19 ± 0.58	0.85–1.55
	Eyes Closed																
LD (*n* = 7)	1.64 ± 0.34	1.33–1.94	1.79 ± 0.31	1.50–2.07	1.72 ± 0.33	0.50–2.95	1.54 ± 0.61	0.62–2.83	LD (*n* = 11)	1.81 ± 0.50	1.48–2.15	1.70 ± 0.27	1.52–1.89	2.23 ± 1.34	1.33–3.13	2.69 ± 1.86	1.32–3.47
HD (*n* = 7)	1.95 ± 0.46	1.53–2.38	1.54 ± 0.44	1.14–1.95	1.72 ± 1.34	0.47–2.97	1.73 ± 1.19	0.99–2.11	HD (*n* = 11)	1.76 ± 0.25	1.59–1.93	2.20 ± 1.00	1.52–2.88	1.46 ± 0.86	0.89–2.04	2.39 ± 1.59	1.44–3.94
	Single Leg Stance																
LD (*n* = 7)	4.06 ± 1.05	2.77–5.36	4.04 ± 0.78	3.08–5.01	4.65 ± 0.94 ^#^	3.48–5.82	4.40 ± 1.36 ^#^	2.71–6.09	LD (*n* = 11)	4.22 ± 1.11	3.48–4.98	4.28 ± 1.15	3.50–5.05	6.84 ± 3.54	4.46–9.22	6.35 ± 3.39	4.07–8.62
HD (*n* = 7)	4.74 ± 1.49	3.36–6.13	4.68 ± 1.22	3.55–5.81	7.64 ± 2.82	5.04–10.26	8.01 ± 4.48	3.87–12.15	HD (*n* = 9)	4.84 ± 1.05	4.04–5.65	4.39 ± 1.05	3.59–5.20	5.71 ± 1.94	4.22–7.21	6.21 ± 2.61	4.21–8.21
	Tandem Stance																
LD (*n* = 7)	2.94 ± 0.65	1.91–3.96	3.33 ± 1.17	1.47–5.18	3.23 ± 0.79	1.97–4.49	3.68 ± 0.71	2.54–4.82	LD (*n* = 12)	3.48 ± 0.95	2.86–4.08	3.07 ± 0.82 *	2.54–3.59	5.56 ± 2.80	3.78–7.34	4.04 ± 2.81	2.25–5.83
HD (*n* = 7)	3.31 ± 1.17	2.08–4.53	3.16 ± 0.68	2.45–3.88	4.09 ± 1.91	2.08–6.10	3.58 ± 1.41	2.10–5.06	HD (*n* = 13)	3.26 ± 0.97	2.67–3.85	2.97 ± 1.05 *	2.33–3.61	4.21 ± 3.81	1.91–6.51	3.93 ± 2.70	2.29–5.56

CoP: Centre of pressure indicator; LD: Vitamin D dose of 800 IU/day, HD: Vitamin D dose of 4000 IU/day, * significant difference before and after particular training at *p* < 0.025, ^#^ significant difference between supplementation doses at *p* < 0.025.
